# From Response to Prevention: Enhancing EMS Impact With Harm Reduction and HIV Education

**DOI:** 10.7759/cureus.68884

**Published:** 2024-09-07

**Authors:** Daryl O Traylor

**Affiliations:** 1 Public Health, A.T. Still University's (ATSU) College of Graduate Health Studies (CGHS), Mesa, USA; 2 Basic Sciences, University of the Incarnate Word School of Osteopathic Medicine, San Antonio, USA

**Keywords:** emergency medical services, ems training, harm reduction, hiv prevention, injection drug use

## Abstract

EMS are crucial not only for immediate life-saving interventions but also for broader public health initiatives, particularly in harm reduction and HIV prevention. However, many EMS training programs lack comprehensive education and training in these areas, resulting in significant gaps in patient care and provider safety. As the opioid epidemic continues to devastate communities, the need for EMS personnel to be trained in harm reduction strategies, such as naloxone administration, and HIV prevention, has become increasingly urgent. Integrating harm reduction and HIV prevention into EMS training is essential for equipping first responders to effectively address the complex needs of individuals affected by addiction. This training is not only vital for improving public health outcomes but also for ensuring the safety and efficacy of EMS providers in their critical roles on the front lines. The evidence strongly supports the immediate inclusion of harm reduction and HIV prevention in EMS curricula to enhance care quality, reduce the spread of HIV, and combat the ongoing opioid crisis.

## Editorial

EMS play a critical role in life-saving care during emergencies like cardiac arrests, severe trauma, and respiratory distress. However, the role of EMS providers extends far beyond immediate medical interventions. EMS providers are also essential players in broader public health efforts, particularly in harm reduction and HIV prevention. Despite this, many EMS training programs inadequately cover these topics, creating dangerous gaps in both patient care and the safety of EMS providers [1]. The absence of structured training on harm reduction and HIV prevention leaves EMS personnel ill-prepared to address the needs of intravenous drug users (IVDUs), who are at high risk for HIV and other bloodborne diseases. This editorial argues for the urgent integration of harm reduction and HIV prevention education into EMS training programs, based on evidence of the benefits and necessity of such training.

The opioid epidemic is one of the most severe public health crises in the United States, with addiction, overdoses, and fatalities ravaging communities nationwide [1]. Opioids, including prescription painkillers and illicit drugs like heroin and fentanyl, have driven an alarming increase in overdose deaths over the past decade [2]. Harm reduction strategies, such as distributing sterile syringes to prevent HIV and hepatitis C transmission, establishing supervised injection sites, and providing naloxone to reverse overdoses, are proven approaches to mitigating this crisis [3,4]. EMS personnel, who are often the first to respond to overdoses, are uniquely positioned to implement these strategies effectively. Yet, without proper training in harm reduction and HIV prevention, their ability to do so is severely limited. Incorporating this training into EMS programs is not just beneficial, it is essential for empowering first responders to meet the complex needs of those affected by addiction and to combat the intertwined opioid and HIV epidemics effectively.

The global prevalence of HIV among IVDUs underscores the necessity of this training. As of 2021, an estimated 13 million people worldwide who inject drugs were living with HIV [5]. In the United States, HIV transmission among IVDUs remains a significant public health challenge [6]. However, inadequate training in harm reduction and HIV prevention has consistently led to poor patient outcomes and increased risks for EMS providers themselves [7-11]. For instance, a study in Australia found that EMS providers' lack of knowledge in these areas directly contributed to suboptimal care and increased their exposure to bloodborne pathogens [11]. Integrating harm reduction and HIV prevention education into EMS training is an urgent necessity for improving public health outcomes and safeguarding EMS personnel.

Comprehensive training in harm reduction would equip EMS providers with the tools and knowledge needed to deliver compassionate, effective care to drug users. Studies have shown that EMS providers trained in harm reduction and naloxone administration exhibit a marked increase in their willingness to implement these lifesaving interventions [12,13]. Furthermore, this training enhances EMS providers' confidence, reduces job-related stress, and improves job satisfaction, all of which contribute to better patient care and outcomes [13]. These benefits highlight the critical importance of prioritizing harm reduction and HIV prevention in EMS education.

Implementing harm reduction programs within EMS, such as naloxone distribution and training, has proven to be a key strategy in addressing the opioid crisis. Examples from Scotland's national naloxone program and initiatives in the United States, like the Guilford County Solution to the Opioid Problem (GSTOP) project, demonstrate the potential impact of these programs [14-16]. Although some initiatives have shown mixed results, the role of EMS in preventing overdoses and connecting patients to long-term treatment options remains vital. Overcoming barriers to these programs, such as funding constraints and resistance to change, is essential for their success and sustainability.

Best practices for integrating harm reduction and HIV prevention into EMS training emphasize the importance of cultural sensitivity and addressing the stigma associated with drug use and HIV [17,18]. Needle exchange programs and supervised injection facilities have proven effective in reducing morbidity and mortality among IVDUs [12]. EMS training should include comprehensive education on substances like fentanyl, effective pain management, and respectful patient communication [12]. Collaboration across public health, law enforcement, and other sectors is also crucial for the successful implementation of harm reduction policies.

The evidence is unequivocal: integrating harm reduction and HIV prevention into EMS training is essential and must be prioritized to enhance the effectiveness and safety of emergency medical care. This training will enable EMS providers to deliver high-quality care to IVDUs, improve public health outcomes related to the spread of HIV, and ensure their own safety. EMS educators and policymakers must act decisively to incorporate these harm reduction and HIV prevention strategies into training curricula. The time for action is now. By prioritizing harm reduction and HIV prevention in EMS training, we can significantly reduce the devastating impact of the opioid and HIV epidemics on our communities.
